# Estimation of Seasonal Influenza Attack Rates and Antibody Dynamics in Children Using Cross-Sectional Serological Data

**DOI:** 10.1093/infdis/jiaa338

**Published:** 2020-06-18

**Authors:** Amanda Minter, Katja Hoschler, Ya Jankey Jagne, Hadijatou Sallah, Edwin Armitage, Benjamin Lindsey, James A Hay, Steven Riley, Thushan I de Silva, Adam J Kucharski

**Affiliations:** 1 Centre for the Mathematical Modelling of Infectious Diseases, London School of Hygiene & Tropical Medicine, London, United Kingdom; 2 Respiratory Virus Reference Department, Public Health England, London, United Kingdom; 3 Medical Research Council Unit The Gambia at the London School of Hygiene & Tropical Medicine, Banjul, The Gambia; 4 MRC Centre for Global Infectious Disease Analysis, Department of Infectious Disease Epidemiology, School of Public Health, Imperial College London, London, United Kingdom; 5 Center for Communicable Disease Dynamics, Department of Epidemiology, Harvard T. H. Chan School of Public Health, Boston, Massachusetts, USA; 6 The Florey Institute, Department of Infection, Immunity and Cardiovascular Disease, Medical School, University of Sheffield, Sheffield, United Kingdom

**Keywords:** influenza, childhood infection, The Gambia, serology, Bayesian model

## Abstract

Directly measuring evidence of influenza infections is difficult, especially in low-surveillance settings such as sub-Saharan Africa. Using a Bayesian model, we estimated unobserved infection times and underlying antibody responses to influenza A/H3N2, using cross-sectional serum antibody responses to 4 strains in children aged 24–60 months. Among the 242 individuals, we estimated a variable seasonal attack rate and found that most children had ≥1 infection before 2 years of age. Our results are consistent with previously published high attack rates in children. The modeling approach highlights how cross-sectional serological data can be used to estimate epidemiological dynamics.

## Teaser

Using cross-sectional serological data with a mathematical model of cross-reactive antibody dynamics, we estimate the timing and frequency of childhood influenza A/H3N2 infections in The Gambia, with almost half of participants infected in the largest annual epidemic.

Influenza epidemics cause substantial global burden [1], with individuals infected with multiple viral strains during their lifetime [2, 3]. In sub-Saharan Africa, seasonal influenza can lead to high mortality rates and a large burden of illness in children [4]. There is evidence that early-life exposure to influenza viruses can shape both subsequent antibody responses to later strains [5] and risk of disease [6]. However, because infections may be asymptomatic or subclinical [7], it can be challenging to measure these early-life infections. Understanding of early life seasonal influenza infections in sub-Saharan Africa is further limited by the relative paucity of seroepidemiological studies of childhood populations.

We tested contemporary serum samples from cross-sectional samples in a childhood cohort from The Gambia against a panel of recently circulating influenza A/H3N2 strains [8]. Combining these hemagglutination inhibition (HI) assays with a Bayesian model of unobserved infections and antibody dynamics [9, 10], we estimated the frequency and timing of infections in the cohort. We then used these results to reconstruct seasonal attack rates, as well as characterize antibody responses in children after primary infections with influenza A/H3N2.

## MATERIALS AND METHODS

### Study Population and Serological Testing

**Figure 1.**
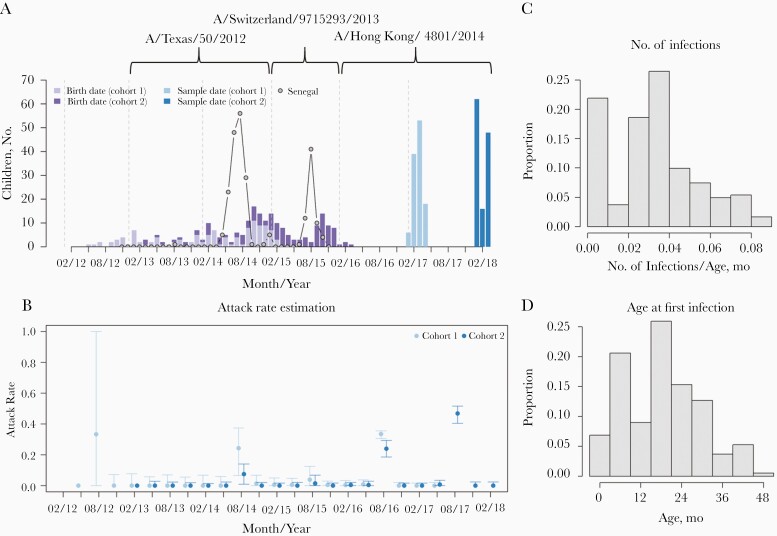
*A,* Number of individuals in each cohort with their respective birth month during 2012–16 and sampling month during 2017–18. Circles indicate frequency of A/H3N2 infections observed in Senegal from 2013 to 2015 (obtained from Niang et al [11]). *B*, Estimated attack rate for the Gambia cohorts with 95% quantile interval. *C,* Estimated number of infections per age in months. *D,* Estimated age at first infection.

Two cohorts of unvaccinated children aged between 24 and 60 months living within a periurban area in The Gambia were recruited between February and April in 2017 (n = 116) and between January and March in 2018 (n = 126), as part of a prospective observational phase 4 study of live attenuated influenza vaccine (Figure 1A; see details elsewhere [8]); a total of 242 children were included in the current study. The median age across both cohorts was 35 months (range, 24–59 months). Prevaccination serum samples were taken from each participant. Each sample was tested by HI assay against a panel of 3 egg-cultured influenza A/H3N2 vaccine viruses: A/Texas/50/2012, A/Switzerland/9715293/2013, and A/Hong Kong/4801/2014. These were chosen to represent the main antigenically distinct A/H3N2 strains circulating during the lifetime of the children, based on World Health Organizations influenza vaccine recommendations for the Northern hemisphere.

### Statistical Analysis

We used a Bayesian inference framework to jointly estimate infection histories and cross-reactive antibody dynamics for each participant [10]. The overall methods are described elsewhere [9], and we detail the specific assumptions of our application in the supplementary information. For an unknown sequence of infection times from year of birth until year of sampling, a mechanistic model of antibody boosting, waning, and cross-reaction generated a predicted antibody titer against each test strain for each participant. The predicted assay response (HI titer) was defined by a normally distributed random variable, with mean equal to the predicted antibody response and standard deviation reflecting assay measurement error; these were interval censored to reflect measured 2-fold HI dilutions (Supplementary Information). The model also estimated a time-varying population-level probability of infection to account for correlation in infection risk between individuals during outbreaks. Using this framework, we estimated infection times and model parameters from the serological data.

We considered infections on a quarterly timescale (ie, individuals could be infected once per 3-month window), with quarters defined as follows: quarter 1, 1 January to 31 March; quarter 2, 1 April to 30 June; quarter 3, 1 July to 30 September; and quarter 4, 1 October to 31 December. We assumed that the antigenic distance between strains increased linearly with time, reflecting the locally linear average path of A/H3N2 antigenic evolution observed in antigenic maps of cross-reactive responses to A/H3N2 derived from naive ferret antiserum [2, 12]. A Euclidean distance of 1 unit on the antigenic map corresponds to a 2-fold reduction in HI titer between the strain that generated the antiserum and the cross-reactive strain being measured. We assumed that there was no detectable antigenic change within a specific calendar year and that strains circulated according to the periods of time they were selected as vaccine components (Figure 1A).

We incorporated prior information about dynamic antibody dynamic in the model. Specifically, the level of short-term rise in titer after infection, the rate of waning of this short-term response, and subsequent persistent level of titer were informed by previous analysis of HI data using the same model [10]. In the absence of any surveillance data from The Gambia, we incorporated prior knowledge about the times of infection based on epidemiological data from neighboring Senegal [11]: we assumed that infection was most likely to occur in the third quarter of the year, with a very low probability of infection in the remaining quarters. In 2013, there was also very little H3N2 influenza infection reported in Senegal [11]; we therefore assumed very little infection probability in all quarters in 2013 (see Supplementary Material for more details). Incorporating these prior assumptions in our model, we inferred the cross-reactive antibody dynamics and individual-level times of infections for children in the cohort, using the “serosolver” package [9]. From these estimates, we calculated the attack rate, as well as the frequency of individual-level infections, predicted antibody responses, and predicted assay responses (HI titers) against different A/H3N2 strains.

## RESULTS

We estimated that influenza A/H3N2 attack rates varied considerably over the study time period. The 2017 attack rate in cohort 2 was highest, with 47% (95% credible interval [CrI], 40%–52%) of children infected. The second highest attack rate was in 2016, with 24% (95% CrI, 19%–29%) of cohort 2 and 33% (95% CrI, 31%–36%) of cohort 1 infected. There were lower attack rates in 2014 and little evidence of infections in 2013 and 2015 (Figure 1B). It was not possible to estimate the attack rate with any confidence in 2012, because few individuals were born at this point.

As well as estimating population-level dynamics, we could compare individual-level infection histories. We estimated that 45% of children sampled had 1 infection in their lifetime. Of those who had been infected, the age at first infection ranged from 0 to 5 years (ie, the age range of the entire cohort), with a median age of 21 months (Figure 1D). Of 242 individuals sampled, we estimated that 33% had >1 infection, with only 1 having evidence of 3 infections. We found that the individuals with more past infections tended to be older (Supplementary Figure 1). The estimated time between infections for the individuals with >1 infection ranged from 1 quarter to 4 years (with 61% having 2 years between infections).

**Figure 2.**
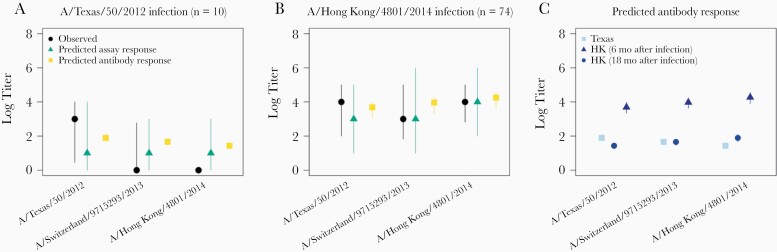
Characteristic antibody profiles for different infection histories. Predicted antibody titers were calculated for each individual, using 200 draws from the posterior estimates of the antibody parameters. Points indicate medians, and vertical lines, 95% quantile intervals. Plots are the observed response (circles), predicted antibody response (no observation error) (squares), and predicted assay response (with observation error) (triangles) to the test strains when individuals were estimated to have had only 1 infection with A/Texas/50/2012 (A) or A/Hong Kong/4801/2014 (B) within a year of sampling. C, Predicted antibody responses for hypothetical individuals sampled in 2018, who were infected with either A/Texas/50/2012 (infection in 2013), A/Hong Kong/4801/2014 within 18 months of the sampling date, or A/Hong Kong/4801/2014 within 6 months of the sampling date.

Individual-level log titers predicted by the model corresponded well with observed log titers (Figure 2A and 2B). Because the model separately accounted for antibody dynamics and measurement error, it was possible to generate predictions for antibody response (true serum antibody levels) and assay response (observed antibody titer) separately. The observed and predicted antibody titers for those individuals infected with A/Texas/50/2012 (Figure 2A) decrease with increasing strain circulation time.

By modeling the underlying antibody dynamics, the model was able to capture the variation in observed log titer according to when participants would have been infected. Participants with an inferred recent infection with A/Hong Kong/4801/2014 (n = 62) (Figure 2B) had higher antibody titers than those infected with A/Texas/50/2012 (n = 8). Although very few individuals were estimated to have been infected with A/Switzerland/9715293/2013, log titers of ≥3 (equivalent to a raw titer of 1:40) were observed for the majority of participants estimated to have had 1 previous infection, which in the model was explained by a high estimated level of cross-reaction.

The effect of time since infection on predicted measurements is illustrated in Figure 2C. For the predicted antibody response, the level of response to an A/Hong Kong/4801/2014 infection 18 months from the sampling date and an A/Texas/50/2012 infection was lower than response to a more recent infection with A/Hong Kong/4801/2014 within 6 months of the sampling date. Our model was able to capture the process of antibody waning with time after infection.

## DISCUSSION

By testing contemporary serum against strains antigenically similar to those circulating during the lifetime of a pediatric cohort, and adjusting for cross-reactive antibody dynamics and assay uncertainty using a Bayesian model, we estimated the epidemiology of childhood influenza A/H3N2 infections in The Gambia. We found high seasonal attack rates in several years, with almost half the population infected in the peak year. Most children had a primary A/H3N2 infection by 2 years of age. Our range of attack rate estimates was broadly consistent with previously published estimates of infection risk in children from observed data; a systematic review of randomized controlled vaccine trials from 32 countries estimated a combined symptomatic and asymptomatic attack rate of 22.5% (95% confidence interval, 9.0%–46.0%) [13].

Our study had some limitations. We imposed a quarterly timescale in our model owing to the observation that neighboring Senegal has a high-risk influenza season focused on a single quarter. Because our data are cross-sectional, without this prior probability assumed for the timing of infection, we would not be able to infer differences between quarters. Although we had strong priors on the timing of infection, we did not enforce a strong prior on the magnitude of infection in the third quarter. Higher-resolution estimates could be possible if multiple serological samples were collected within each year. In addition, infection in this study is measured as HI response; there are other assays and forms of testing that may confirm infection in individuals we have reported as negative.

We also assumed that circulating influenza viruses corresponded to the vaccine strain at the time, and that for A/Texas/50/2012 and A/Hong Kong/4801/2014, the same strain circulated for 2 consecutive years. Although seasonal influenza viruses can emerge many months ahead of the vaccine strain selection, epidemics were concentrated in a small portion of the year in our model, so the broad sequence of antigenic change observed in A/H3N2 viruses in The Gambia during 2011–18 is unlikely to be substantially different from that assumed. For viruses with well-described egg adaptations, including A/Switzerland/9715293/2013 and A/Hong Kong/4801/2014, we anticipate that HI titers would be smaller in magnitude than if the viruses tested were more similar to the actual circulating virus. This would mean that our inferred attack rates are lower than the true attack rates.

Previous studies have found that longitudinal data are required to reliably estimate waning antibody responses [3]. We therefore incorporated prior information on the relative magnitudes of short- and long-term antibody responses, with a strong prior on the rate of waning. However, our final estimates for the boost in titer after infection were generally higher than the prior estimate, suggesting sufficient information in the data to estimate child-specific magnitudes of response (Supplementary Figure 2 and Supplementary Figure 3). We assumed a common waning rate for both adults and children. In reality, this rate may be different in children and adults, but in the absence of a robust estimate for children only, we chose to use an inferred waning rate from a mixed-age cohort. Previous studies have shown that in mixed-age cohorts that the waning rate is close to a year; hence, it is likely that within the timescales we are using any differences in waning rate would not make a substantial difference to inferred attack rates. Our attack rates estimates were robust to prior information on waning only (Supplementary Figure 4). We also incorporated prior information about the timing of infection within a year, with the low attack estimated rate in 2013 (median, 0 in both cohorts) informed by Senegal surveillance data [11]. In the absence of this prior information in 2013, there was little difference in the estimated attack rates (Supplementary Figure 5).

Early-life infections are important for influenza but have been historically challenging to measure. Applying modeling frameworks to data generated by testing contemporary serum samples tested against multiple historical strains opens up the possibility of estimating epidemiological dynamics in a wide variety of settings. Using our methods, this could be achieved through either new serological surveys or secondary analysis of existing serum banks [14]. This may be especially important in countries where there is a paucity of influenza surveillance and incidence data, yet where details of influenza attack rates are vital for planning public health policy around influenza prevention. In addition to providing insight into population- and individual-level risk, knowledge of unobserved prior infections could be a useful predictor variable in analysis of subsequent infection risk, disease risk, or responses to vaccination, given the increasing evidence of immune imprinting during childhood on these parameters [6].

## Supplementary Data

Supplementary materials are available at *The Journal of Infectious Diseases online.* Consisting of data provided by the authors to benefit the reader, the posted materials are not copyedited and are the sole responsibility of the authors, so questions or comments should be addressed to the corresponding author.

jiaa338_suppl_Supplementary_Figure_1

jiaa338_suppl_Supplementary_Figure_2

jiaa338_suppl_Supplementary_Figure_3

jiaa338_suppl_Supplementary_Figure_4

jiaa338_suppl_Supplementary_Figure_5

jiaa338_suppl_Supplementary_Material

## Notes

***Financial support.*** This work was supported by a Sir Henry Dale Fellowship jointly funded by the Wellcome Trust and the Royal Society (206250/Z/17/Z) to A. J. K.; Wellcome Trust Intermediate Clinical Fellowship award (110058/Z/15/Z) to T. I. S.; Medical Research Council (MR/R015600/1) to J. A. H. and S. R. and Wellcome Trust Investigator Award (200861/Z/16/Z) to S. R.

***Potential conflicts of interest.*** All authors: No reported conflicts. All authors have submitted the ICMJE Form for Disclosure of Potential Conflicts of Interest. Conflicts that the editors consider relevant to the content of the manuscript have been disclosed.
