# Supplementary figures and images for: Estimation of Seasonal Influenza Attack Rates and Antibody Dynamics in Children Using Cross-Sectional Serological Data
Source: J Infect Dis. 2020 Jun 18;225(10):1750–4. doi: 10.1093/infdis/jiaa338 (PMC9113438; doi:10.1093/infdis/jiaa338)

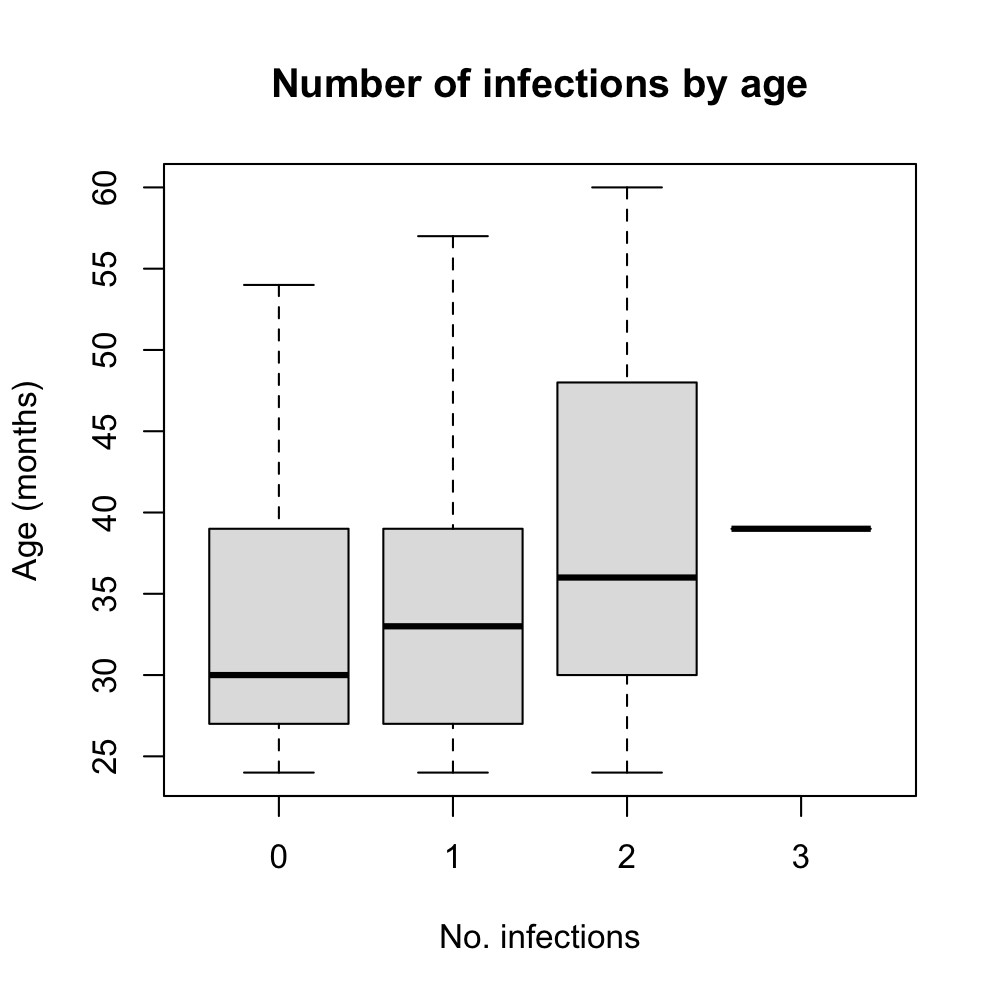

Supplement: jiaa338_suppl_Supplementary_Figure_1 [file jiaa338_suppl_supplementary_figure_1.jpeg]

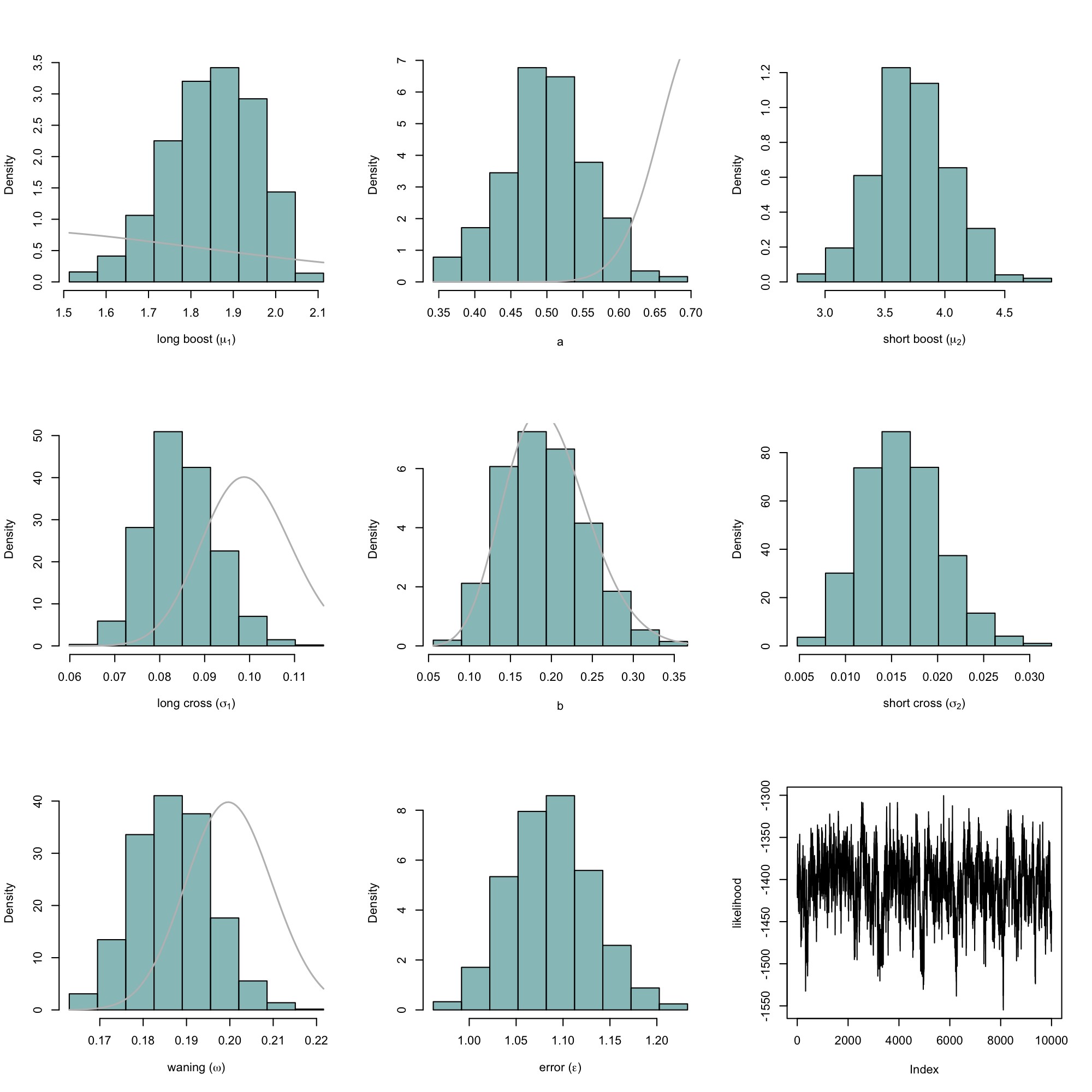

Supplement: jiaa338_suppl_Supplementary_Figure_2 [file jiaa338_suppl_supplementary_figure_2.jpeg]

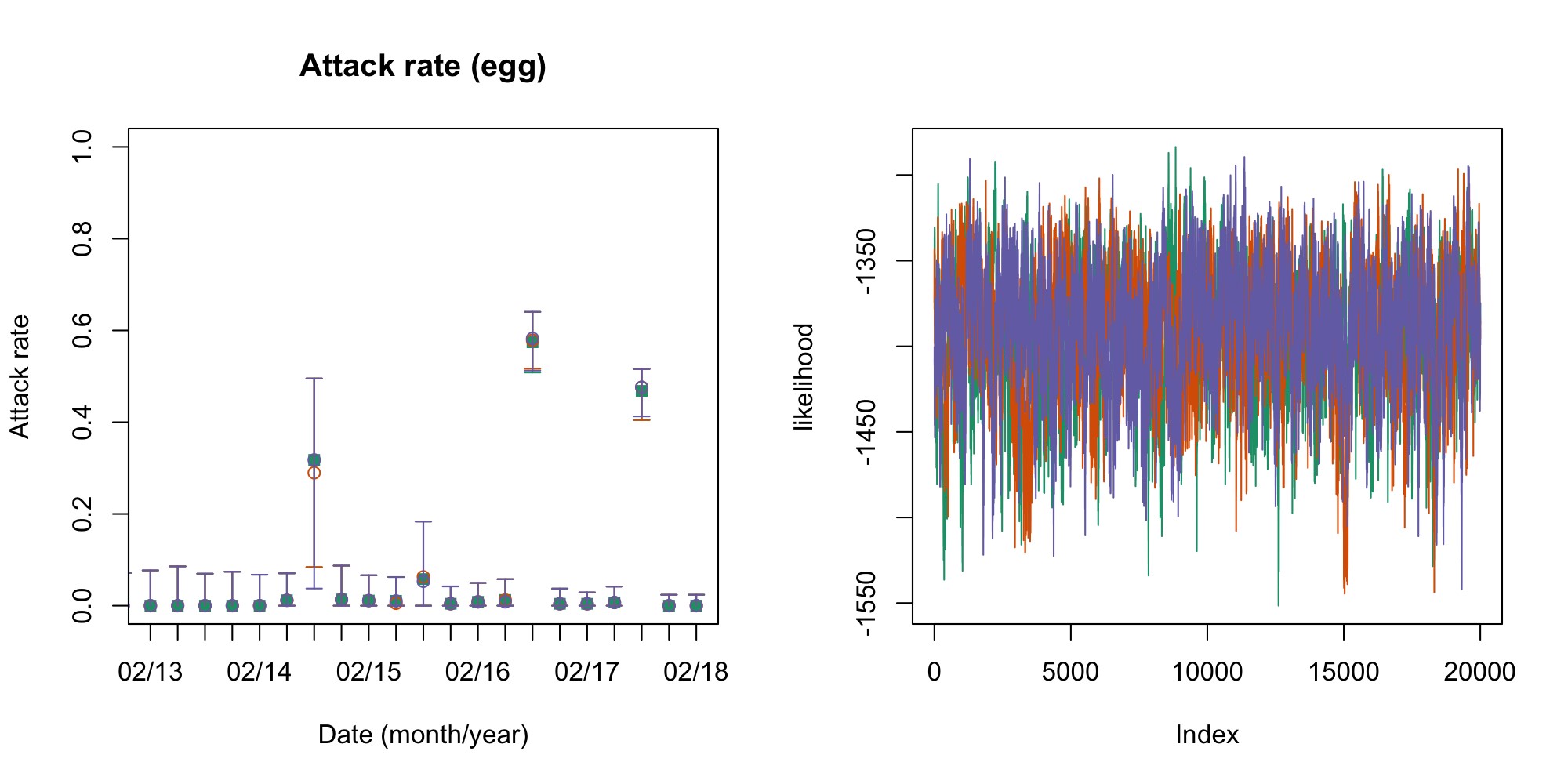

Supplement: jiaa338_suppl_Supplementary_Figure_3 [file jiaa338_suppl_supplementary_figure_3.jpeg]

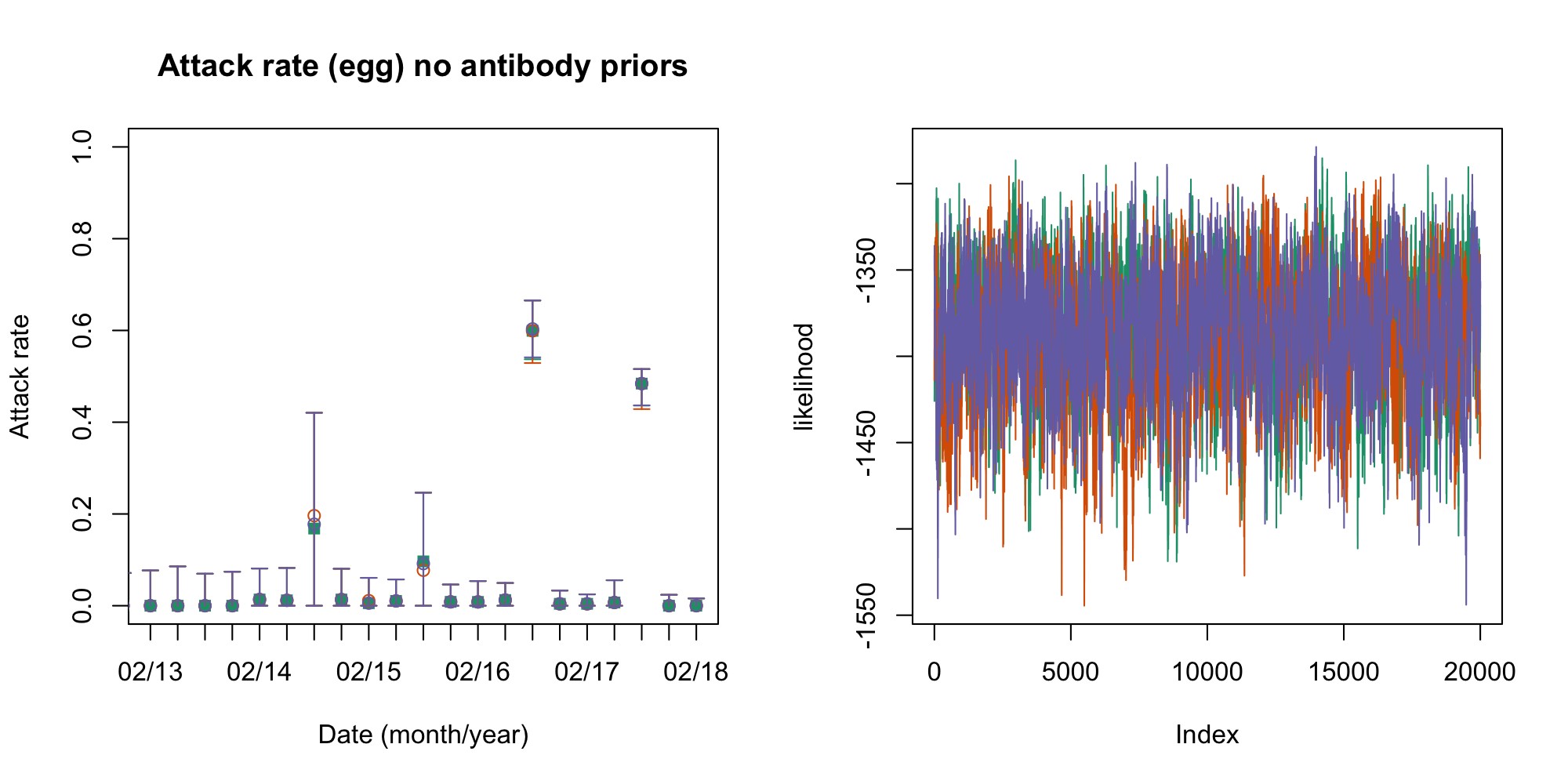

Supplement: jiaa338_suppl_Supplementary_Figure_4 [file jiaa338_suppl_supplementary_figure_4.jpeg]

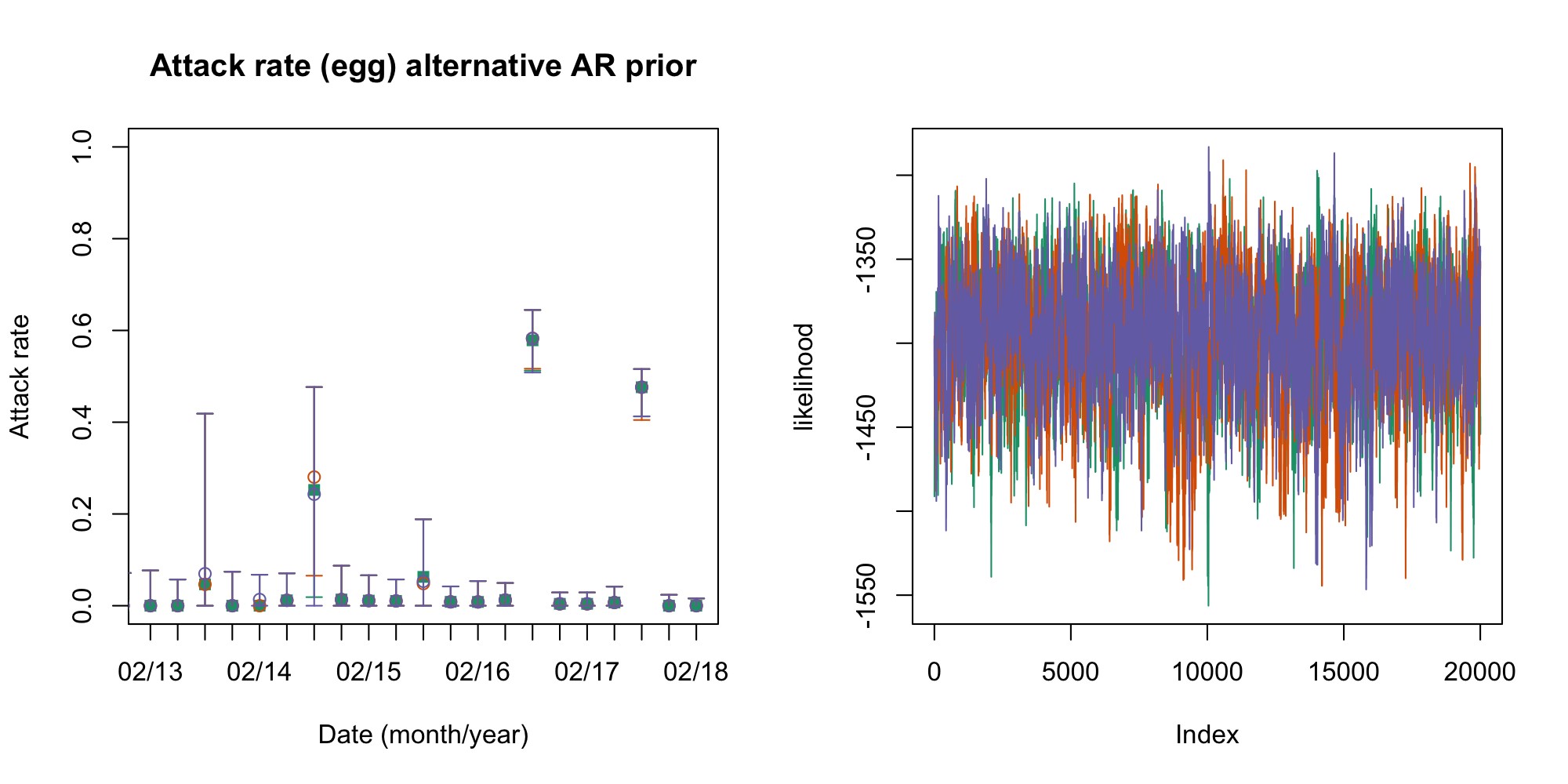

Supplement: jiaa338_suppl_Supplementary_Figure_5 [file jiaa338_suppl_supplementary_figure_5.jpeg]
