# Supplementary material for: Estimation of Seasonal Influenza Attack Rates and Antibody Dynamics in Children Using Cross-Sectional Serological Data
Source: J Infect Dis. 2020 Jun 18;225(10):1750–4. doi: 10.1093/infdis/jiaa338 (PMC9113438; doi:10.1093/infdis/jiaa338)
Supplement: jiaa338_suppl_Supplementary_Material [file jiaa338_suppl_supplementary_material.pdf]

Supplementary information

Ethics Statement

This study was approved by The Gambia Government and UK Medical Research Council (MRC) joint ethics committee and the Medicines Control Agency of The Gambia.

Modelling antibody titre

We infer infection times by mathematically modelling the underlying antibody response individuals have to different test strains. We define the infection history for each individual i, $\boldsymbol{Z_i}$, as a vector of binary latent states for the presence or absence of infection at each time point. The expected log-titre for an individual i against a strain circulating at time j and observed at time t was a linear combination of antibody responses from each prior infection,

$$X_{i,j,t} = \sum_{k \in \boldsymbol{Z_i}} Z_{i,k} \left(\mu_l d_l(j,k) + \mu_s w(t,k) d_s(j,k) \right) \tag{1}$$

where μ_l, μ_s are the long- and short-term boosts of the antibody response. The short-term boost linearly wanes over time at a rate ω. The long- and short-term boosts are scaled down according to the long- and short-term level of cross reaction $d_l()$, $d_s()$ between the infecting strain (k) and the test strain (j). Cross-reactivity was modelled as $d_{l,s}(j,k) = \max(0, 1 - \sigma_{l,s}\delta_{k,j})$ where $\delta_{k,j}$ was the two-dimensional antigenic distance between strains k and j and $\sigma_{l,s}$ are the long- and short-term cross-reaction parameters to be fitted. Here, the long-term boost is specified as a function of the short term boost as follows $\mu_l = a\mu_s$ where a is bounded between 0 and 1. Similarly, we specify the short term cross-reaction term as $\sigma_s = b\sigma_l$ where b is bounded between 0 and 1.

We assume that the strains that circulated during our study period reflect those in the World Health Organisation (WHO) influenza vaccine recommendations for the Northern hemisphere (Table S1) and that within these circulation periods there was no detectable change in antigenic distance.

We used the inference framework described in [1] to infer the time of infection and

Table S1. Details of assumptions surrounding the circulating A/H3N2 influenza viruses during the lifetime of children recruited into the study. Viruses were assumed to have circulated in seasons when the World Health Organisation (WHO) influenza vaccine recommendations for the Northern hemisphere included that virus.

Season circulated	Virus name
2013-2014	A/Texas/50/2012
2014-2015	A/Texas/50/2012
2015-2016	A/Switzerland/ 9715293/2013
2016-2017	A/Hong Kong/ 4801/2014
2017-2018	A/Hong Kong/ 4801/2014

underlying antibody parameters, where infections occur conditional on a population level infection probabilities $\mathbf{\Phi}$, which can be specified per time unit. In particular we implemented hyper-priors on the probability of infection in each time period to reflect the seasonality of infection.

The analysis was performed using the R package serosolver (https://github.com/seroanalytics/serosolver). The specific case study code can be found under the branch alternative parameterisation as well as a subset of anonymised data.

Priors

We assumed some prior knowledge of the distribution of antibody process parameters (Table S2). The long term boost, waning and cross-reaction prior distributions were informed by independent model fits from data sets of HI titres from [2].

Table S2. Model parameters and prior distributions.

Parameter	Prior Distribution
Long term boost (μ_l)	Gamma(shape = 9, rate = 6). Mean = 1.5
Waning (ω)	Beta(shape1 = 318, shape2 = 1272). Mean = 0.2
a	Beta(shape1 = 57,shape2 = 24)
Long term cross-reaction (σ_l)	Beta(shape1 = 90, shape2=813). Mean = 0.1.
b	Beta(shape1 = 12, shape2 = 49)

In each time unit, in this case quarters, there is an explicit probability of infection. We incorporate prior knowledge of the time of infection by assuming that most infection occurs in the third quarter of the year, Uniform(0, 1), and very little of infection in the remaining quarters, Beta(shape1 = 1, shape2 = 50). This is with the exception of 2013, in which there was very little influenza infection reported in Senegal [3]. We assume that the infection dynamics in Senegal reflect those in Gambia, and so include prior knowledge of very little infection probability in all quarters of 2013. Using these priors and this method we infer time of infection and antibody

process parameters using the Metropolis within Gibbs scheme presented by [1]. From these measures we can calculate epidemiological measures of interest.

Posteriors

Using the inferred time of infections, we calculated the age at first infection for individuals in the cohort. Figure S1 shows that age distribution for different estimated numbers of infections. Those individuals with the highest number of infections also were the oldest at the time of sampling.

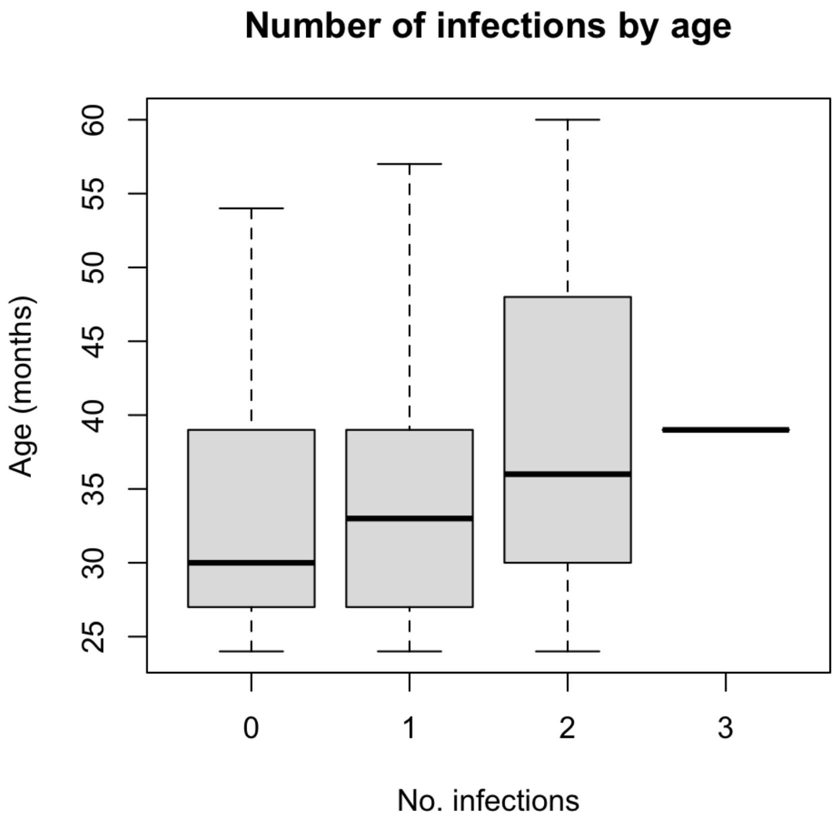

Fig S1. Age of participants by estimated number of infections.

The posterior distributions for the antibody process parameters for the titre data, where applicable the prior distribution density has been added in grey are shown in Figure S2.

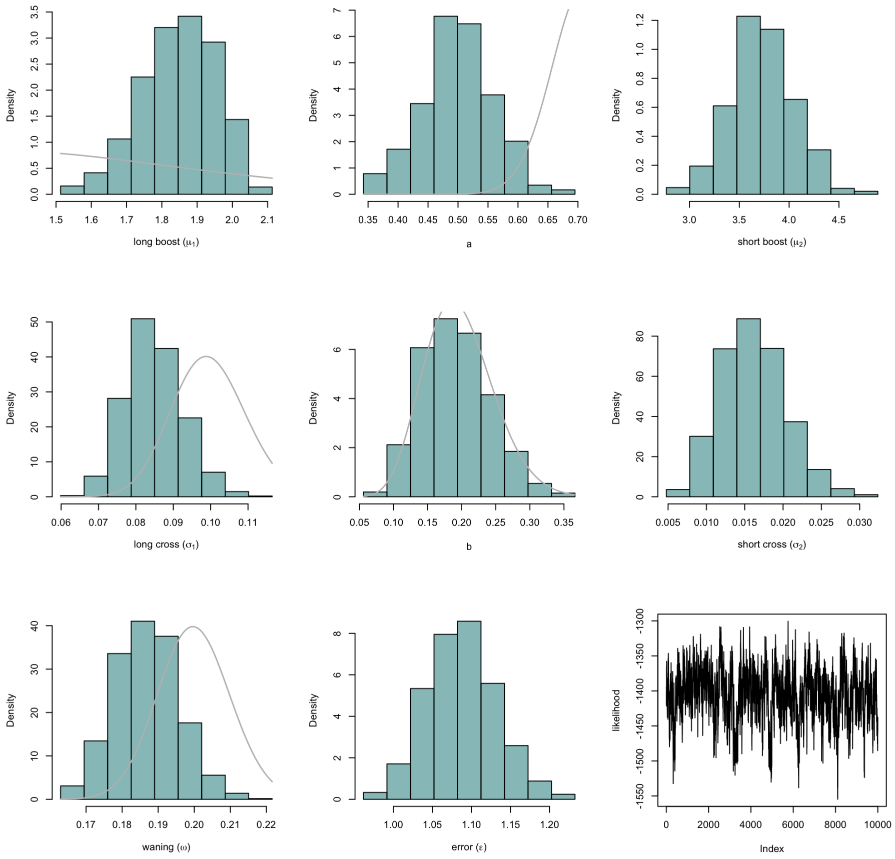

Fig S2. Posterior densities of parameters and their prior distributions in grey.

Sensitivity analyses

For each sensitivity run, three chains were run and the Gelman-Rubin diagnostic was calculated for the antibody process parameters. In all cases, the upper limit of the confidence interval was less than 1.1, indicating that the chains had converged. For the main result, we ran three separate chains to confirm convergence (Figure S3).

Antibody parameter prior information

In the main report and in Figure S2 we implemented prior distributions informed by previously published studies. With cross-sectional data, the waning rate of the antibody response cannot be estimated but it is of interest to understand how robust our attack rate estimates are to less prior information on the remaining antibody parameters. The estimated attack rates and likelihood chain plots in Figure S4 with prior information on the waning only, were similar to the estimated attack rates with all prior information incorporated.

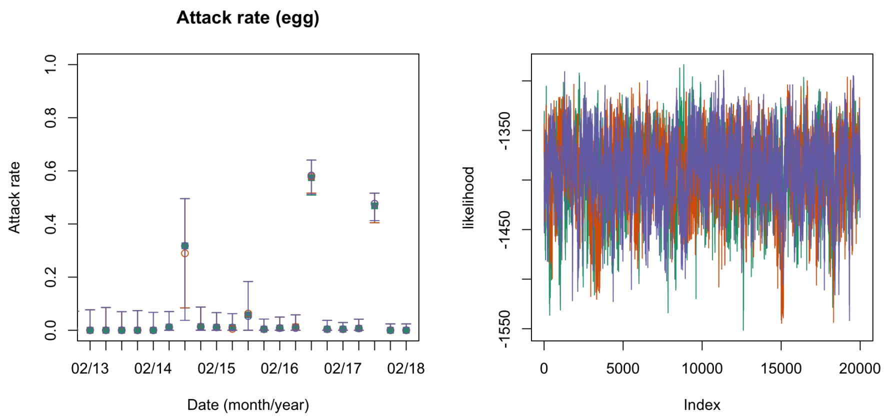

Fig S3. Estimated attack rate for the Gambia cohorts with 95% quantile interval of three different MCMC chains using titre response and the corresponding trace plot of the likelihood values of three chains.

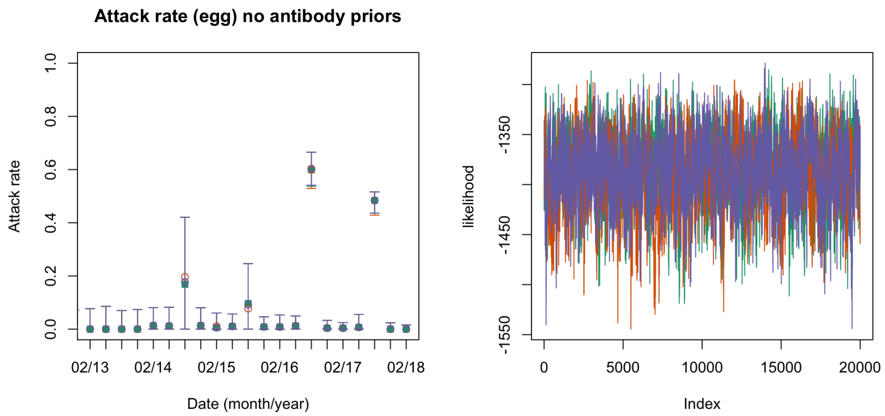

Fig S4. Estimated attack rate for the Gambia cohorts with 95% quantile interval of three different MCMC chains using titre response with waning only prior distribution.

Lower attack rate in 2013

Due to the low incidence of A/H3N2 observed in Senegal in 2013 [3], we imposed prior information that the 3rd quarter in 2013 had a low attack rate. In the absence of this additional prior information, we see that the estimated attack rates in 2013 has a low median but a much wider range (Figure S5), and that the attack rates were similar in all other time points.

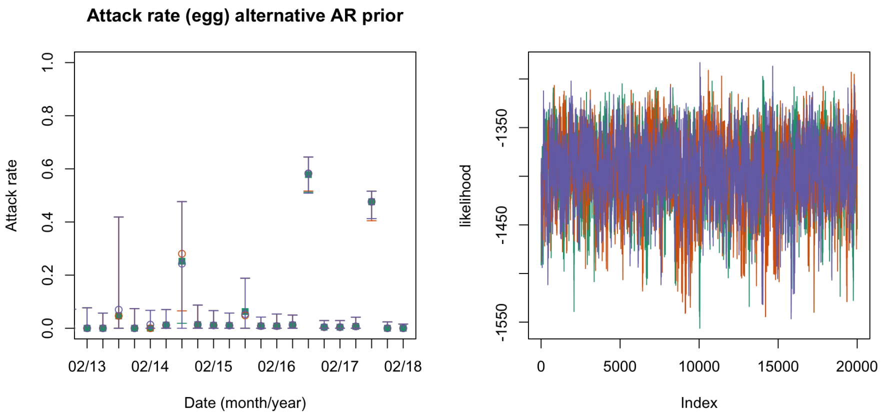

Fig S5. Estimated attack rate for the Gambia cohorts with 95% quantile interval of three different MCMC chains using titre response when the lower prior in 2013 was not imposed.

References

[1] Hay JA, Minter A, Ainslie K, Lessler J, Kucharski AJ, Riley S. Serosolver: an open source tool to infer epidemiological and immunological dynamics from serological data. bioRxiv. 2019;.

[2] Kucharski AJ, Lessler J, Cummings DAT, Riley S. Timescales of influenza A/H3N2 antibody dynamics. PLoS Biol. 2018 08;16:1–19.

[3] Niang MN, Barry MA, Talla C, Mbengue A, Sarr FD, Ba IO, et al. Estimation of the burden of flu-association influenza-like illness visits on total clinic visits through the sentinel influenza monitoring system in Senegal during the 2013 - 2015 influenza seasons. Epidemiol Infect. 2018;146(16):2049–2055.
